# The epigenetic landscape of age-related diseases: the geroscience perspective

**DOI:** 10.1007/s10522-017-9695-7

**Published:** 2017-03-28

**Authors:** Noémie Gensous, Maria Giulia Bacalini, Chiara Pirazzini, Elena Marasco, Cristina Giuliani, Francesco Ravaioli, Giacomo Mengozzi, Claudia Bertarelli, Maria Giustina Palmas, Claudio Franceschi, Paolo Garagnani

**Affiliations:** 1 Department of Experimental, Diagnostic and Specialty Medicine, University of Bologna, Via San Giacomo 12, Bologna, 40126 Italy; 2 IRCCS Institute of Neurological Sciences, Bologna, Italy; 3 Department of Biological Geological and Environmental Sciences, Laboratory of Molecular Anthropology & Centre for Genome Biology, University of Bologna, Bologna, Italy; 4 Interdepartmental Center “L. Galvani”, University of Bologna, Bologna, Italy; 5 Center for Applied Biomedical Research (CRBA), St. Orsola-Malpighi University Hospital, Bologna, Italy; 6 Clinical Chemistry, Department of Laboratory Medicine, Karolinska Institutet at Huddinge University Hospital, S-141 86 Stockholm, Sweden

**Keywords:** Aging, Age-related diseases, Epigenetics, DNA methylation

## Abstract

In this review, we summarize current knowledge regarding the epigenetics of age-related diseases, focusing on those studies that have described DNA methylation landscape in cardio-vascular diseases, musculoskeletal function and frailty. We stress the importance of adopting the conceptual framework of “geroscience”, which starts from the observation that advanced age is the major risk factor for several of these pathologies and aims at identifying the mechanistic links between aging and age-related diseases. DNA methylation undergoes a profound remodeling during aging, which includes global hypomethylation of the genome, hypermethylation at specific loci and an increase in inter-individual variation and in stochastic changes of DNA methylation values. These epigenetic modifications can be an important contributor to the development of age-related diseases, but our understanding on the complex relationship between the epigenetic signatures of aging and age-related disease is still poor. The most relevant results in this field come from the use of the so called “epigenetics clocks” in cohorts of subjects affected by age-related diseases. We report these studies in final section of this review.

Aging is a multifactorial and complex process, characterized by a gradual decline in physiological functions, associated with decreased fitness and higher risk of major diseases. Recent conceptualizations have proposed a set of pillars, that is processes that promote aging in a highly interconnected way (Mahmoudi and Brunet 2012; López-Otín et al. 2013; Kennedy et al. 2014). Among these pillars, epigenetic mechanisms have emerged as playing a major role in aging and became an important subject of aging biology research.

DNA methylation, on which this review will focus on, is one of the most studied and best-characterized epigenetic modifications which substantially changes during aging. It consists in the covalent addition of a methyl group to the carbon-5 position of cytosine residues in CpG dinucleotides in genomic DNA (Jones 2012; Bergman and Cedar 2013). The bulk of the genome is markedly depleted in CpG sites, which on the contrary they are enriched in specific tracts of the genome, named CpG islands, often associated with gene promoters and regulatory regions. The methylation status of CpG islands and surrounding regions (CpG island shores) is an important factor in regulating chromatin structure and gene expression during development and differentiation (Deaton and Bird 2011). DNA methylation is strongly affected by environmental factors, including diet (Bacalini et al. 2014; Johnson and Belshaw 2014; Barrès and Zierath 2016), pollution (Lillycrop and Burdge 2014; Cao 2015) and stress (Gassen et al. 2016). Throughout lifespan, profound alterations in DNA methylation patterns occur (Weidner and Wagner 2014; Zampieri et al. 2015). Specifically, during aging, as reported by data obtained from several human tissues, three different types of changes occur. First, global DNA methylation level decreases with age. Global hypomethylation occurs principally in repetitive DNA sequences and is considered associated with increased activation of transposable elements (Alexeeff et al. 2013). In addition to global hypomethylation, aging results in differential methylation (mainly hypermethylation) of certain genomic loci, consistently observed within large human cohorts (Maegawa et al. 2010; Rakyan et al. 2010; Grönniger et al. 2010; Hernandez et al. 2011; Bocker et al. 2011; Bell et al. 2012; Heyn et al. 2012; Beerman et al. 2013; McClay et al. 2014; Reynolds et al. 2014; Bacalini et al. 2015). Finally, with advanced age, it is observed an increase in inter-individual divergence between patterns of DNA methylation, referred as epigenetic drift (Fraga et al. 2005; Bjornsson et al. 2008; Talens et al. 2012; Yuan et al. 2015; Slieker et al. 2016), and in stochastic epigenetic mutations (Gentilini et al. 2015).

A large body of evidence indicates that age-dependent DNA methylation remodelling is largely recapitulated in cancer (Baylin and Jones 2011; Klutstein et al. 2016) and, according to several authors, epigenetic changes that occur during physiological aging can increase the risk of tumor onset and progression (Lin and Wagner 2015; Klutstein et al. 2016). Paradoxically, while the intimate relationship between the epigenetic changes that occur in aging and those that occur in cancer has been largely discussed, this conceptual framework has been largely neglected in the study of other age-related diseases. It is well established that aging increases susceptibility to many diseases and it is considered as the largest risk factor for some of the most prevalent illness in Western countries. Nevertheless, as we will show in this review, most of the studies that have investigated the epigenetic landscape of age-related diseases accurately described the loci in which DNA methylation changes occur, but did not discuss their relationship with the patterns characteristic of physiological aging. This new approach in the study of age-related diseases is part of the “geroscience” conceptual framework (Kennedy et al. 2014), which highlights the large interconnection between aging and age-related diseases and aims to systematically identify the mechanistic cellular and molecular connections between the two phenomena. In the epigenetics field, the “aging perspective” in the study of age-related diseases has been appreciated only recently thanks to availability of the so-called “epigenetic clocks” (Bocklandt et al. 2011; Horvath 2013; Hannum et al. 2013; Weidner et al. 2014; Snir et al. 2016), that is mathematical models calibrated on the DNA methylation changes that occur during physiological aging. As we will show, samples from subjects suffering of age-related diseases tend to be epigenetically older than healthy individuals according to these clocks, suggesting that age-related diseases are characterized by the acceleration of the DNA methylation changes that normally occur during physiological aging.

In the following sections, we will discuss current knowledge on the DNA methylation patterns of age-related diseases and their relationship with age-specific patterns, focusing on cardio-vascular diseases (CVD), musculoskeletal function and frailty.

## Epigenetics and age-related cardiovascular diseases

Atherosclerosis, CVD and their complications are a major cause of mortality and morbidity. Their incidence and prevalence dramatically increase with age, and aging is considered as a major intrinsic cardiovascular risk factor. Impact of epigenetic mechanisms in cardiovascular physiopathology represents one of the hottest research topics of the last years. Numerous risk factors of CVD have been identified since many years and role of DNA methylation, by its position at the interface between genes and environment, has been deeply investigated. Thus, regarding dyslipidaemia, epigenome-wide association studies (EWAS) have been carried out, identifying genes with different methylation profiles among affected individuals and controls. Carnitine palmitoyltransferase-1A (*CPT1A*) gene has emerged from the comparison of CD4 + T cells isolated from a large cohort of individuals (991 subjects from the GOLDN study): methylation at some specific CpG sites, located in intron 1 of *CPT1A,* have been found to be associated with very-low density lipoprotein (VLDL), low-density lipoprotein (LDL) and triglycerides (TG) blood parameters (Frazier-Wood et al. 2014; Irvin et al. 2014). Association between DNA methylation at the identified CpG sites and TG profile was replicated in the Framingham Heart Study (Irvin et al. 2014). *CPT1A* plays an important role in maintaining a healthy lipid profile, by its involvement in mitochondrial β-oxidation of long chain fatty acids. An increase in its expression may be associated with a favourable lipid profile, but it is still not clear whether an increase in DNA methylation determines a higher or lower *CPT1A* expression level. This gene has also emerged from another study, where the association between blood lipid levels and whole blood DNA methylation has been evaluated on 1776 subjects from another cohort (Pfeiffer et al. 2015). In this study, *CPT1A* gene product has been found to interact with 2 other proteins, encoded by ABC-transporter G1 (*ABCG1*) and sterol regulatory element-binding transcription factor 1 (*MIR33B/SREBF1*) genes. *SREBF1* enhances lipogenesis by activating the synthesis of fatty acids and cholesterol, and its expression is simultaneous to that of *MIR33B* (Marquart et al. 2010). Activation of this pathway can determine impairment of both fatty acids metabolism and cholesterol homeostasis.

Some other genes have been found differentially methylated between individuals with dyslipidaemia and controls. For example, in a cohort of patients with familial hypercholesterolaemia, hypermethylation of the promoter region of ATP-binding cassette A1 (*ABCA1*), which encodes a protein involved in cholesterol transfer from blood to high-density lipoprotein (HDL) particles, was found to be associated with lower levels of HDL-cholesterol and with prior history of coronary artery disease (CAD) (Guay et al. 2012). Furthermore, methylation of apolipoprotein E (*APOE*) was recently found to be associated with total cholesterol plasma levels (Ma et al. 2015).

Unbalanced lipid accumulation establishes a vicious circle in which chronic inflammation of blood vessels walls gives rise to immune activation that, in turn, foster inflammation and immune cells recruitment, leading to the luminal narrowing characteristic of atherosclerosis (Weber and Noels 2011). As regards atherosclerosis, an EWAS on matched healthy and atherosclerotic human aortic samples was carried out and a specific epigenomic profile has been identified (Zaina et al. 2014). This atherosclerotic signature includes genes involved in crucial processes such as, among others, activity of endothelial and vascular smooth muscle cells (SMCs), monocytes migration, angiogenesis, blood coagulation and atherosclerotic plaque instability and size. Along with previous results, in atherosclerotic lesions, both a global hypermethylation of Alu sequences (Kim et al. 2010) and a reduction of the expression level of enzymes that mediate active DNA demethylation (Liu et al. 2013) were detected (Zaina et al. 2014). Recently, the same research group identified specific CpG loci that undergo DNA methylation drift towards hypermethylation in parallel to the atherosclerosis lesions progression (del Pilar Valencia-Morales et al. 2015), reporting this way a possible promising epigenomic marker of CVD disease.

Of particular interest in atherosclerosis is the study of the methylation status of estrogen receptor-genes (*ER*) whose protective functions have been described for cardiovascular system (Mendelsohn and Karas 1999). Methylation of *ESR*-α gene was found to be increased in vascular specimens displaying atherosclerotic lesions (Post et al. 1999) and it was also observed a same pattern of DNA methylation changes in SMCs displaying a proliferative phenotype compared to cells originated from non-atherosclerotic aortas (Ying et al. 2000). Migration and proliferation of SMCs are involved in vascular injury and importance of another gene, named collagen type XV alpha 1 (*COL15A1*), has emerged from in vitro studies on proliferating SMCs. COL15A1 is an extracellular matrix protein that can modulate the SMCs phenotype, acting on their ability to proliferate and migrate. Interestingly, methylation level of *COL15A1* gene changes according to the number of replications of SMCs (Connelly et al. 2013). A passage-dependent DNA methylation increase was also reported for *ER*-β gene, that was found to be hypermethylated when plaque and non-plaque regions were compared (Kim et al. 2007). The observed hypermethylation trend with increasing replicative passage number is similar to that that may occur physiologically with aging.

Regarding CAD, one of the most common consequence of atherosclerosis, hypermethylation of specific DMRs was observed in an EWAS performed in patients with ischemic cardiopathy (Sharma et al. 2014). Some of these DMRs are located in genes important for cardiovascular system, including *C1QL4*, *CCDC47*, transforming growth factor, beta receptor III (*TGFBR3*), *ADA*, *HAND2* and *IKIP*. In particular, *TGFBR3* plays a crucial role in maintaining cardiovascular health, as it inhibits *TGF*-*β1* signalling, acting this way as an anti-fibrosis factor (Liang et al. 2012). Interestingly, a treatment with simvastatin up-regulates *TGFBR3* expression, limiting this way cardiac fibrosis, by suppressing ERK1/2 and JNK-dependent fibroblast activity and collagen production (Sun et al. 2015). Otherwise, *ADA* has been found to be involved in the control of inflammation hypertension-dependent mediated by physical activity (Cardoso et al. 2015). Genetic variants within this gene are associated to the pathogenesis of chronic heart failure (He et al. 2014) and CAD (Safranow et al. 2007; Saccucci et al. 2014). Finally, *HAND2* is a cardiomyocyte marker, involved in cardiogenesis and coronary vasculogenesis (VanDusen et al. 2014) as well as cardiovascular system development (Dirkx et al. 2013).

There is a growing body of evidence that environment and lifestyle, i.e. diet, smoking, alcohol consumption or physical activity can modulate cardiovascular risk by changing DNA methylation profiles. As an example, it has been recently demonstrated that DNA methylation can mediate the health benefits gained from regular physical exercise training (Denham et al. 2015): a group of 12 healthy young men underwent a sprint interval training three times a week and genome-wide leukocyte DNA methylation was measured before and after 4 weeks. A general demethylation was observed in CpG islands and in gene promoters, suggesting a corresponding change in the genomic expression profile. Particularly interesting is the demethylation of epidermal growth factor (*EGF*) gene, associated with a decreased mRNA expression. This can result in an anti-atherogenic effect, since it can limit the activity of EGF receptor that mediates inflammation and vessel damage (Makki et al. 2013).

## Epigenetics and musculoskeletal function during aging

### Sarcopenia

Musculoskeletal system is greatly affected during aging, leading to decreased mobility. Adult skeletal muscle (SM) is characterized by a progressive loss of mass, strength and functionality. This condition, caused by the replacement of SM with adipose and fibrotic tissue and by the decrease in the regenerative capacity, is known as sarcopenia (Walston 2012). Progressive accumulation of non-contractile tissue results in functional impairment and physical disability in the elderly. Different molecular mechanisms, including epigenetics, regulate maintenance of muscle mass and sarcopenia could result from a dysregulation of these processes.

Regenerative capability of SM tissue is effected by satellite cells, the muscle stem cells located in niche, between basal lamina and sarcolemma (Yin et al. 2013). In physiological conditions, SM is characterized by a low-grade proliferation and in the absence of stimuli from the microenvironment, satellite cells are quiescent. However, they are able to escape from this state and rapidly proliferate and differentiate in response to injury or other stressors. Quiescence has long been viewed as a dormant state, but recent insights indicate an actively and strictly control of quiescence to maintain a poised state that permits rapid activation (Cheung and Rando 2013). Regenerative process in adult muscle, initiated by injury, stress or pathological signals, is activated through the paired box protein 7 (Pax7), a transcription factor that engage the myogenic program for new-myofibers generation (Kassar-Duchossoy et al. 2005; Relaix et al. 2006). Age-related changes of somatic stem cells cause an unbalanced between quiescent/proliferative cell number, causing a decline in their regenerative capability (Jung and Brack 2014). Epigenetic alterations in some crucial signaling pathways, like fibroblast growth factor receptor-1 (FGFR1), JAK/STAT or p38 MAPK, have been observed in satellite cells from aged mice (Segalés et al. 2016), contributing in compromised stem cell function. Thus, epigenetic changes seem to profoundly affect the function of stem cells and contribute to functional degradation (Pollina and Brunet 2011).

Of particular interest are the results of the first EWAS conducted on human SM, published by Zykovich et al. in 2014. Samples collected from healthy older adults were compared to younger ones (Zykovich et al. 2014). Authors identified several differential methylated CpG nucleotides between younger and older tissues and selected 500 CpG sites which were able to discriminate younger subjects from older ones. Aged muscle showed hypermethylation mainly in intragenic sequences (middle and 3′ end of genes), rather than in the promoter regions. Ontology analysis on intragenic regions methylation changes during aging revealed that the most enriched pathways were muscle functions and neuromuscular junctions.

Another environmental cue that could influence DNA methylation in SM cells is physical exercise, a factor that can maintain muscle mass and a better autonomy in older people. According to observational studies, mainly performed using blood samples, the effect appears moderate. However, some interventional studies examined specifically the impact of training on DNA methylation status of muscle tissue obtained by muscular biopsies. After acute training, it was observed a decreased in whole genome methylation (Barrès et al. 2012). Hypomethylation of promoters was associated with an increased expression of some genes involved in energy metabolism or mitochondrial function especially, such as peroxisome proliferator-activated receptor gamma coactivator 1-alpha (*PGC*-*1*α), pyruvate dehydrogenase kinase isoenzyme 4 (PDK4), or peroxisome proliferator-activated receptor γ (PPAR-γ), in a dose-dependent manner. These results suggest that DNA methylation changes can represent an active and adaptive response to exercise. More recently, Lindholm et al. performed an EWAS in a prospective study, in order to analyze methylation changes in human SM with training (Lindholm et al. 2014). To avoid any confounding factors, authors obtained an intra-individual control, training only one leg per subject. Through this innovative approach, they observed significantly modulation in DNA methylation after long-term endurance exercise training (3 months). Substantial increase of methylation level occurred mainly in regulatory and enhancer regions and was associated with a coordinated transcriptional response. Interestingly, modifications in methylation levels seemed to be a precise process, occurring in genes involved in structural changes of muscle tissue, inflammation and immunological pathways. It should be noted that those studies were mostly performed on young and healthy adults and that, to date, no interventional study examined effects of training on DNA methylation status of SM cells in elderly people.

Interestingly, changes in genome-wide DNA methylation patterns after exercise were also observed in SM from individuals who are first-degree relative of patients with type 2 diabetes (Nitert et al. 2012): authors noticed epigenetic differences in muscle from patients who are at risk for diabetes (relative to their family history), as compared to muscle from patients who are not at risk for this disease. Genes differentially methylated are involved in muscle biological pathways, such as insulin, MAPK or calcium signaling. After 6 months of exercise, like in the other studies cited, epigenetics changes were observed, with a reduction in the number of genes which were found to be differentially methylated between the two groups of subjects (38 post-intervention versus 65 genes prior).

### Osteoarthritis

Osteoarthritis (OA) is a comorbidity frequently observed in the elderly. This degradation of articular cartilage is associated with chronic disability, increased risk of fall and loss of independence.

During the last 3 years, results of EWAS conducted on OA cartilage samples, in different centres, were reported (Delgado-Calle et al. 2013; Fernández-Tajes et al. 2014; Jeffries et al. 2014): these studies identified large numbers of differentially methylated CpG sites in OA cartilages, compared to controls [healthy controls (Fernández-Tajes et al. 2014) or osteoporotic patients (Delgado-Calle et al. 2013)]. Genes that were found to be differentially methylated were previously identified as implicated in OA pathogenesis by their roles in skeletal/cartilage development and inflammatory responses. It was also reported that, within the same joint, there is a significant differential CpG methylation between matched eroded or intact cartilage (Jeffries et al. 2014), whereas subchondral bone seemed to share the same epigenetic phenotype as overlying eroded cartilage (Jeffries et al. 2016). Methylation levels of some CpG sites on OA cartilage were correlated with the histopathologic disease severity score, assessed by modified Mankin score (Jeffries et al. 2014). Recently, Zhang et al. reported changes in DNA methylation profile of OA chondrocytes according to disease progression, with changes preferentially observed at last stage of OA (Zhang et al. 2016).

## Epigenetics and frailty

Frailty is a clinical geriatric syndrome associated with an increased vulnerability following stressors (Chen et al. 2014). Phenotypic definition of frailty includes different criteria, such as weakness, slowness, low level of physical activity, exhaustion and weight loss. Frail and pre-frail states are common among older adults. They are complex states, resulting from the combination of poor physiologic reserves and weakness. They are associated with an increased risk of falls, disability, hospitalization and mortality (Fried et al. 2001). Sarcopenia, described above, is associated and contributes greatly to the frailty syndrome. These two conditions represent a crucial health problem for the elderly.

Impact of changes in DNA methylation occurring during aging on frailty has been poorly specifically investigated. A first study showed that frail individuals, aged between 65 and 89 years old, exhibited lower global DNA methylation levels, compared to subjects who were pre-frail or non-frail (Bellizzi et al. 2012). A 7-years follow-up study, dealing with 37 subjects, revealed that a worsening in the frailty status was associated with significant decrease in global DNA methylation level. In 2014, Collerton et al. investigated, in a cohort of 321 subjects aged 85 years, associations between frailty and alterations in DNA methylation, both at global and specific levels. 21.8% of the subjects were frail and 58.6% pre-frail. Subjects who presented lower levels of DNA methylation at promoter specific CpG sites had decreased odds of frailty. Unlike the previous study, genome-wide methylation was not correlated with the frailty status (Collerton et al. 2014).

## Epigenetic clock and association to morbidity and mortality

As mentioned above, during aging, hypermethylation and hypomethylation events, involving specific CpG sites, occur and are consistent between individuals. Some sites are highly associated with age and, using data obtained by EWAS, mathematical models were developed to identify potential biomarkers of aging, referred as epigenetic clock. Based on age-related changes in DNA methylation from multiple CpG sites across the genome, it is possible to accurately predict the biological age of an individual (Bocklandt et al. 2011; Horvath 2013; Hannum et al. 2013; Weidner et al. 2014; Snir et al. 2016). Thus, positive deviation of biological age compared to chronological age is considered as an age acceleration. Epigenetic clock results are informative of biological age of individuals, and therefore possibly associated to their health status and to potential occurrence of age-related health outcomes. At least four studies demonstrated that epigenetic clock in blood can predict all-cause mortality (Marioni et al. 2015a; Perna et al. 2016; Christiansen et al. 2016; Chen et al. 2016), even after taking consideration of a variety of other potential confounding variables, including adjustment for blood cell composition. Moreover, it is associated with specific morbidity, such as CVD risk (Perna et al. 2016). Recently, biological age of patients with stroke was determined and compared to controls of the same chronological age: patients with ischemic stroke were biologically older than healthy controls. This was particularly true for younger patients (Soriano-Tárraga et al. 2016). Regarding OA, it was recently shown that cartilages of patients with OA demonstrate a specific accelerated aging as compared to controls, without differences in epigenetic aging of bone nearby and without a significant accelerated epigenetic aging in blood cells of OA patients (Vidal-Bralo et al. 2016). DNA methylation age correlates also with cognitive and physical illness: in a study involving 1091 older individuals, Marioni et al. observed that higher age acceleration was associated with lower abilities, such as fluid cognitive ability, grip strength or lung function (Marioni et al. 2015b). Finally, DNA methylation age was also found to be associated with frailty, assessed by an index corresponding to the proportion of deficits present among 34 items (Breitling et al. 2016).

## Conclusion

Epigenetic changes have been identified as key players in normal and pathological aging and accurate description of the epigenetic landscape of age-related diseases has been provided in the last years. Nevertheless, our understanding of how age-associated epigenetic changes contribute to the epigenetic dysregulation and, more in general, to the pathology of age-related diseases is still at the beginning. The new conceptual framework of the “geroscience” (Kennedy et al. 2014) has focused the need to systematically investigate the links between aging and age-related pathologies, in order to understand how age enables disease. Currently, the most relevant result in this field is the acceleration in DNA methylation age observed in age-related diseases using Horvath’s and Hannum’s epigenetic clocks. This observation points for an intimate connection between epigenetic dysregulation in physiological and pathological aging, but it is often difficult to dissect the single components of these algorithms in order to identify the most relevant genomic loci affected by aging and potentially associated with the diseases. Therefore, future studies should explicitly investigate the existence of an overlap between DNA methylation signatures in chronological age and in age-related diseases, as it has been demonstrated for cancer (Lin and Wagner 2015), and identify those genomic loci that undergo epigenetic changes during chronological aging and that are also clearly affected in age-related diseases. Finally, future EWAS studies on age-related diseases should take into account not only the relationship between the observed epigenetic signatures and those characteristic of aging, but also the complex interaction with the (internal and external) environment, that can shape the epigenetic identity of each individual in different ways during life course. Lessons come from the field of genetics of age-related diseases, where it has been theorized and demonstrated that the association of a genetic variant with a disease can vary during the life of an individual and according to the environmental conditions that he/she encountered, strictly depending from the birth cohort (Franceschi et al. 2000; Ukraintseva et al. 2016; Franceschi and Garagnani 2016; Kulminski et al. 2016). Moreover, the study of the epigenetics of age-related diseases will certainly benefit from the use of longitudinally followed cohorts, which allow to take into account the complex relationship between age and pathologies. In conclusion, we foresee that the implementation of a geroscience perspective in the study of the epigenetics of age-related diseases will shed light into the mechanisms of the pathology and will pave the way for new therapeutical approaches, based on pharmacological, nutritional or physical interventions, that counteracting aging will counteract also age-related diseases.
